# Vitiligo-specific instrument on quality of life - Brazilian Portuguese version*

**DOI:** 10.1590/abd1806-4841.20165744

**Published:** 2016

**Authors:** Juliana Catucci Boza, Natália Piccinini Giongo, Tania Ferreira Cestari

**Affiliations:** 1 Universidade Federal do Rio Grande do Sul (UFRGS) – Porto Alegre (RS), Brazil

Mr. Editor,

As the authors of the article "Translation, cross-cultural adaptation and validation of the vitiligo-specific health-related quality of life instrument (VitiQoL) into Brazilian Portuguese", we would like to publish the translated version of VitiQol in Portuguese (Chart 1 and 2).^1^ This instrument has only been published in English, and we have been contacted by other health professionals to provide a validated version in Portuguese.

**Chart 1:** VitiQoL PB

*The purpose of these questions is to assess whether the aspect of your skin affected your life during the last month.*								
DURING THE LAST MONTH, from 0 (not at all/ not applicable) to 6 (very much):								
	**1.** *Did you feel bothered by the appearance of your skin?* **2.** *Did you feel frustrated about your skin condition?* **3.** *Did you feel difficulty in showing affection because of your skin condition* ? **4.** *Has your skin condition affected your daily activities?* **5.** *While talking to someone, did you worry about what people might be thinking about you?* **6.** *Were you afraid that people would criticize you?* **7.** *Did you feel embarrassed or inhibited because of your skin?* **8.** *Did the appearance of your skin influence your choice of clothing?* **9.** *Did your skin condition affect your social or leisure activities?* **10.** *Did your skin condition affect your emotional well-being?* **11.** *Did your skin condition affect your physical health (as a whole)?* **12.** *Did your skin condition influence your care with personal appearance (for instance, haircut or use of cosmetic products)?* **13.** *Did your skin condition influence your sun protection care during leisure activities (for instance, limiting the time of exposure to* * the sun during peak hours, staying out of the sun, wearing a hat, long sleeves or pants)?* **14.** *Did your skin condition affect the possibility of making new friends?* **15.** *Did you worry about disease progression to other body parts?* **16.** *Please rate how severe you think your skin condition is:*							
**Severity of skin condition**								

	**0**	**1**	**2**	**3**	**4**	**5**	**6**	


Reproduced with permission of the authors ALL RIGHTS RESERVED © Lilly E, Kundu RV 2012. Any unauthorized use or reproduction of this document is strictly prohibited.

**Chart 2:** VitiQoL-PB

*O objetivo destas questões é avaliar quanto a sua pele o afetou durante o último mês.*								
DURANTE O ÚLTIMO MÊS, de 0 (nada/ não se aplica) a 6 (muito):								
	**1.** *Você se sentiu incomodado pela aparência do seu problema de pele?* **2.** *Você se sentiu frustrado com o seu problema de pele?* **3.*** Você sentiu dificuldade em demonstrar afeto por causa do seu problema de pele?* **4.** *O seu problema de pele afetou suas atividades diárias?* **5.** *Ao conversar com alguém, você se preocupou com o que poderiam estar pensando de você?* **6.** *Você teve medo de que as pessoas o criticassem?* **7.** *Você se sentiu envergonhado ou inibido por causa da sua pele?* **8.** *O seu problema de pele influenciou o tipo de roupa que você usa?* **9.** *O seu problema de pele afetou suas atividades sociais ou de lazer?* **10.** *O seu problema de pele afetou o seu bem-estar emocional?* **11.** *O seu problema de pele afetou a sua saúde física como um todo?* **12.** *O seu problema de pele influenciou os seus cuidados com a aparência pessoal (por exemplo, corte de cabelo ou uso de cosméticos)?* **13.** *O seu problema de pele influenciou os seus cuidados com a proteção solar durante os momentos de lazer (por exemplo, limitação* * do tempo de exposição durante as horas de pico do sol, busca por sombra ou uso de chapéu, mangas compridas ou calças)?* **14.** *O seu problema de pele afetou a possibilidade de você fazer novos amigos?* **15.** *Você se preocupou com a progressão da sua doença para novas áreas de seu corpo?* **16.*** Por favor, avalie quão grave você sente o seu problema de pele:*							
***Gravidade do problema de pele***								

	**0**	**1**	**2**	**3**	**4**	**5**	**6**	


Reproduzido com permissão dos autores TODOS OS DIREITOS RESERVADOS © Lilly E, Kundu RV 2012. Interdita a reprodução, ainda que parcial, do presente documento..

Brazil has insufficient data on the life quality of vitiligo patients. After the English version publication of the vitiligo questionnaire, and once the Brazilian Portuguese version is published, the impact of vitiligo on the quality of life might be assessed by a specific instrument in Brazil.^1,2^ Therefore, it will yield a more complete and reliable evaluation of patients both in medical offices and in new clinical trials.
